# 

**Published:** 1906-07

**Authors:** 


*Sixty-first Year.*

VOL. LXI. JULY, 1906. NO. 12

### BUFFALO MEDICAL JOURNAL

Established 1845 by AUSTIN FLINT, M. D.

LIBRARY
INDEXED
S.G.O.
JUL 12 1906

EDITOR:
WILLIAM WARREN POTTER, M. D.
ASSISTANT EDITORS:
WILLIAM C. KRAUSS, M. D. NELSON W. WILSON, M. D.
ASSOCIATE EDITORS:
JAMES WRIGHT PUTNAM, M. D. ERNEST WENDE, M. D.
JOHN PARMENTER, M. D. JOHN A. MILLER, PH. D.
HARVEY R. GAYLORD, M. D. MAUD J. FRYE, M. D.
JOHN A. RAFTER, M D.

